# LONELINESS AND QUALITY OF LIFE IN OLDER ADULT PRIMARY CARE PATIENTS

**DOI:** 10.1093/geroni/igad104.1993

**Published:** 2023-12-21

**Authors:** Monica Williams-Farrelly, Matthew Schroeder, Claudia Li, Nicole Fowler

**Affiliations:** Indiana University School of Medicine, Indianapolis, Indiana, United States; Regenstrief Institute, Inc., Indianapolis, Indiana, United States; Indiana University School of Medicine, Indianapolis, Indiana, United States; Indiana University School of Medicine, Indianapolis, Indiana, United States

## Abstract

Loneliness, defined as the perceived discrepancy in an individual’s desired and actual social relationships, is common among older adults. Loneliness among older adult primary care patients is lacking, considering the implications it has on physical and mental health. Our objective was to determine the relationship between loneliness and quality of life (QOL) in older adult primary care patients. Data come from the Caregiver Outcomes of Alzheimer’s Disease Screening (COADS) study, an ongoing randomized controlled trial evaluating benefits and risks of Alzheimer’s disease and related dementias screening among older primary care patients and their family members. Loneliness (5-item NIH Toolbox), quality of life (QOL)—as measured by physical and mental health component scores— and depression (PHQ-9) and anxiety symptomatology (GAD-7) were measured among primary care patients aged 65 and older from April 2020 to September 2021. Spearman correlation analyses reveal that loneliness was moderately correlated with mental health (r(601) = -.43, p< 0.001), anxiety (r(601) =.44, p< 0.001), and depression (r(601) = .42, p< 0.001), while weakly correlated with physical health (r(601) = -.15, p< 0.001). After conducting unadjusted and adjusted linear regression models, we found that loneliness was associated with both lower mental (p< 0.001) and physical health component scores (p< 0.001). Furthermore, loneliness remained significantly associated with worse mental health when adjusting for depression, anxiety, sociodemographic characteristics, and comorbidity. Primary care providers should discuss loneliness with their older adult patients and provide resources to help patients develop and maintain meaningful social relationships.

